# Anti-Peroxynitrite Treatment Ameliorated Vasorelaxation of Resistance Arteries in Aging Rats: Involvement with NO-sGC-cGKs Pathway

**DOI:** 10.1371/journal.pone.0104788

**Published:** 2014-08-12

**Authors:** Lu Ma, Ke Wang, Jianyu Shang, Chengzhang Cao, Panpan Zhen, Xin Liu, Wen Wang, Hui Zhang, Yunhui Du, Huirong Liu

**Affiliations:** 1 Department of Physiology and Pathophysiology, School of Basic Medical Sciences, Capital Medical University, Beijing, PR China; 2 Department of Chest Surgery, First Hospital of Longyan, Fujian Medical University, Fujian, PR China; 3 Department of Pathology, Luhe Hospital, Capital Medical University, Beijing, PR China; 4 Beijing Key Laboratory of Metabolic Disturbance Related Cardiovascular Disease, Beijing, PR China; Thomas Jefferson University, United States of America

## Abstract

Declined vasorelaxation function in aging resistance arteries is responsible for aging-related multiple organ dysfunctions. The aim of the present study is to explore the role of peroxynitrite (ONOO^-^) in aging resistance arterial vasorelaxation dysfunction and the possible mechanism. In the present study, young (3–4 months olds) and aging (20 months olds) male SD rats were randomized to receive vehicle (Saline) or FeTMPyP (ONOO^-^ scavenger) for 2 weeks. The vasorelaxation of resistance arteries was determined in vitro; NOx level was tested by a colorimetric assay; the expression of nitrotyrosine (NT), soluble Guanylate Cyclase (sGC), vasodilator-stimulated phosphoprotein (VASP), phosphorylated VASP (P-VASP) and cGMP in resistance arteries were detected by immunohistochemical staining. In the present study, endothelium-dependent dilation in aging resistance arteries was lower than in those from young rats (young *vs.* aging: 68.0%±4.5% *vs.* 50.4%±2.9%, *P*<0.01). And the endothelium-independent dilation remained constant. Compared with young rats, aging increased nitrative stress in resistance arteries, evidenced by elevated NOx production in serum (5.3±1.0 nmol/ml vs. 3.3±1.4 nmol/ml, P<0.05) and increased NT expression (P<0.05). ONOO^-^ was responsible for the vasorelaxation dysfunction, evidenced by normalized vasorelaxation after inhibit ONOO^-^ or its sources (*P*<0.05) and suppressed NT expression after FeTMPyP treatment (*P*<0.05). The expression of sGC was not significantly different between young and aging resistance arteries, but the cGMP level and P-VASP/VASP ratio (biochemical marker of NO-sGC-cGKs signaling) decreased, which was reversed by FeTMPyP treatment in vivo (*P*<0.05). The present study suggested that ONOO^-^ mediated the decline of endothelium-dependent vasorelaxation of aging resistance arteries by induction of the NO-sGC-cGKs pathway dysfunction.

## Introduction

Aging is an independent risk factor for cardiovascular diseases, and aging-related decline of vascular function contributes to both the vascular diseases and multiple organ dysfunctions. Considerable evidence exists that the vasorelaxation function of both aorta [1] and small arteries, such as basilar arteries [2] and coronary arteries [3], decreases with aging. The vasorelaxation dysfunction of resistance arteries plays a crucial role in hypertension and disorder of local blood flow distribution. Moreover, vasodilatation dysfunction in aging resistance arteries contributes to damage of body organs [4]. Nevertheless, mechanisms of aging-related resistance arterial vasodilatation dysfunction remain largely unknown.

The molecular mechanisms of aging-associated vasodilatation dysfunction are complex, which include the elevated endothelin-1 [5], over activated rein-angiotensin system [6] and decreased Nitric Oxide (NO) bioavailability [7]. NO bioavailability decreasing is the primary cause of vasorelaxation dysfunction in aging resistance arteries. In the resistance arteries, NO binds to the heme cofactor of sGC and increases the production of cGMP, which induces the activation of cGMP-dependent protein kinases (cGKs) and vasorelaxation. NO is an essential relaxing factor in NO-sGC-cGKs pathway, and the declined content of NO leads to vasorelaxation dysfunction in aging resistance arteries. However, evidence exists that the production of NO is raised in sera [8] and mesenteric arteries [9] from aging rats.

NO destruction is one of the causes for reduced NO bioavailability in aging arteries [10]. Superoxide is an important detrimental factor in NO destruction. Superoxide anion (**^.^**O_2_^-^) reacts with NO to form peroxynitrite (ONOO^-^) in aging resistance arteries [11]. The reaction consumes NO and produces ONOO^-^. ONOO^-^ is known as a main harmful factor of nitrative stress in many organs. ONOO^-^ impairs cell function in many ways, including the injury to the cell signaling [12]. Several studies reported that endogenous NO-induced vasorelaxation was decreased in arteries from both aging human [13] and rats [14]. It suggests that the NO-related cell signaling is impaired in aging arteries.

The purposes of this study were to determine whether ONOO^-^ was responsible for vasorelaxation dysfunction in aging resistance arteries; and if so, to investigate whether the NO-sGC-cGKs signaling pathway may be involved in ONOO^-^-induced vasorelaxation dysfunction in aging resistance arteries.

## Materials and Methods

### Animals

The investigations conformed to the "Guiding Principles in the Use and Care of Animals" published by the National Institutes of Health (NIH Publication No. 85–23, Revised 1996) and were approved by the Institutional Animal Care and Use Committee of Capital Medical University. Animals were purchased from Dongchuang animal provider, Changsha, Hunan Province, China. Before the experiments, the rats were maintained in 12 hour light-dark cycles and fed ad libitum. Young (3–4 months, 250–300 g) and aging (20 months, 650–750 g) male SD rats were randomly divided into 4 groups: (1) young, (2) young+FeTMPyP (ONOO^-^ scavenger, Cayman), (3) aging, (4) aging+FeTMPyP. Young or aging rats received vehicle (Saline) or FeTMPyP (ONOO^-^ scavenger; 3 mg/kg/3 days; Cayman) via caudal vein injection for 2 weeks. Animals were euthanized by a physical method (decapitation, a suggested method for rodents by *AVMA Guidelines on Euthanasia*). Animals were given chloral hydrate (300 mg/kg) to reduce animals' anxiety on the guillotine, and ensure the euthanasia is rapidly accomplished to less the animals' suffer.

### Blood pressure and heart rate measurement

Blood pressure and heart rate of rats were measured indirectly by using a non-invasive tail-cuff blood pressure measure system (Softron) in awake rats.

### Measurement of resistance arterial vasorelaxation

Small mesenteric arteries are generally considered as the resistance arteries [15]. In the present study, vasorelaxation of third or fourth branches of mesenteric arteries in rats were detected. Freshly harvested intestines were placed into ice-cold and oxygenated Hepes buffer (mM: NaCl, 144; KCl, 5.8; MgCl_2_•6H2O, 1.2; CaCl_2_, 2.5; Glucose, 11.1; Hepes, 5; pH 7.38–7.40). Small mesenteric arteries were isolated (2 mm in length), and mesenteric artery segments were attached to two wires (40 µm) connected to an isometric force transducer (DMT610M, Danish Myo Technology). The segments were suspended in organ bath chambers containing oxygenated (95% O_2_, 5% CO_2_) Hepes buffer. The temperature was set at 37 degrees Celsius. The artery segments were stretched step by step until an optimal resting tension of 100 mmHg, which was maintained throughout the experiment. The segments were equilibrated for 2 h before vasorelaxation measurements were taken.

After the equilibration period, the artery segments were exposed to Hepes buffer containing 60 mM potassium (mM: 89.8, 144; KCl, 60; MgCl2•6H2O, 1.2; CaCl2, 2.5; Glucose, 11.1; Hepes, 5; pH 7.38–7.40) until reproducible contractile responses were obtained. After washing with Hepes buffer, segments of mesenteric arteries were precontracted with norepinephrine (NE, 10^−5^ mol/L). Once a stable contraction was achieved, increasing concentrations of vasodilators (10^−9^-10^−5^ mol/L) were added to the chamber to obtain cumulative concentration-response curves. Endothelium-dependent dilation was measured by Acetylcholine (ACh), and endothelium-independent dilation was measured by sodium nitroprusside (SNP) or S-Nitroso-N-Acetyl-Dl-Penicillamine (SNAP). FeTMPyP (10^−5^ mol/L)[16], Tempol (**^.^**O_2_^-^ scavenger; 10^−4^ mol/L; Sigma Aldrich)[17] or 1400W (inducible nitric oxide synthase (iNOS) inhibiter; 10^−6^ mol/L; Sigma Aldrich)[18] was preincubated in the chamber for 30 min in order to inhibit ONOO^-^ or its sources in young and aging resistance arteries.

### Nitrite plus nitrate measurement

The plasma levels of nitrite plus nitrate (NOx) were assessed as nitrite concentration as described previously [10]. Briefly, serum from young or aging rats was diluted in three times, and ultrafiltered through a 10 kDa cutoff filter (BioVision) to remove serum proteins. NOx was measured by a commercial kit (BioVision) based on the Griess reaction. The nitrate was conversed to nitrite with nitrate reductase. The Griess Reagent was added to the total nitrite, and the color was developed for 10 min at room temperature. After the reaction was completed, NOx concentrations were determined at an optical density of 540 nm by comparison with standard solutions of sodium nitrite.

### Detecting the protein expression by immunohistochemical staining

Immunohistochemical staining was determined using the method as described previously [19]. Briefly, mesentery and mesenteric arteries were removed and stored in 4% paraformaldehyde for <48 h. Fixed mesenteric arteries were dehydrated and embedded in paraffin, and sections were cut into 6 µm thickness and mounted onto glass slides. Antigen was retrieved by using a microwave method (citric acid buffer, PH6.0). Endogenous catalase was inactivated with 3% hydrogen peroxide for 10 min at room temperature. The sections were stained with primary antibody (anti-soluble Guanylate Cyclase (sGC) α1 subunit, Abcam; anti-soluble Guanylate Cyclase (sGC) β1 subunit, Sigma Aldrich; anti-nitrotyrosine (NT), Abcam; anti-vasodilator-stimulated phosphoprotein (VASP), Cell Signaling Technology; anti-phosphorylated VASP (P-VASP), Cell Signaling Technology; cGMP, Merck Millipore) at 4 degrees Celsius overnight and peroxidase-conjugated affinipure secondary antibody (Santa Cruz) at 37 degrees Celsius for 30 min, successively. Target proteins were detected with diaminobenzidine (DAB). Protein quantification was performed by mean density of staining of the vessel tissues using Image Pro (version 6.0).

### Statistics analysis

Statistics analysis was performed with SPSS software (version 13.0). Statistical analysis was carried out by the one-way ANOVA between means of groups. P value <0.05 was considered significant.

## Results

### Vasorelaxation function decreased in aging resistance arteries; and NOx level increased in serum from aging rats

**Figure 1:**
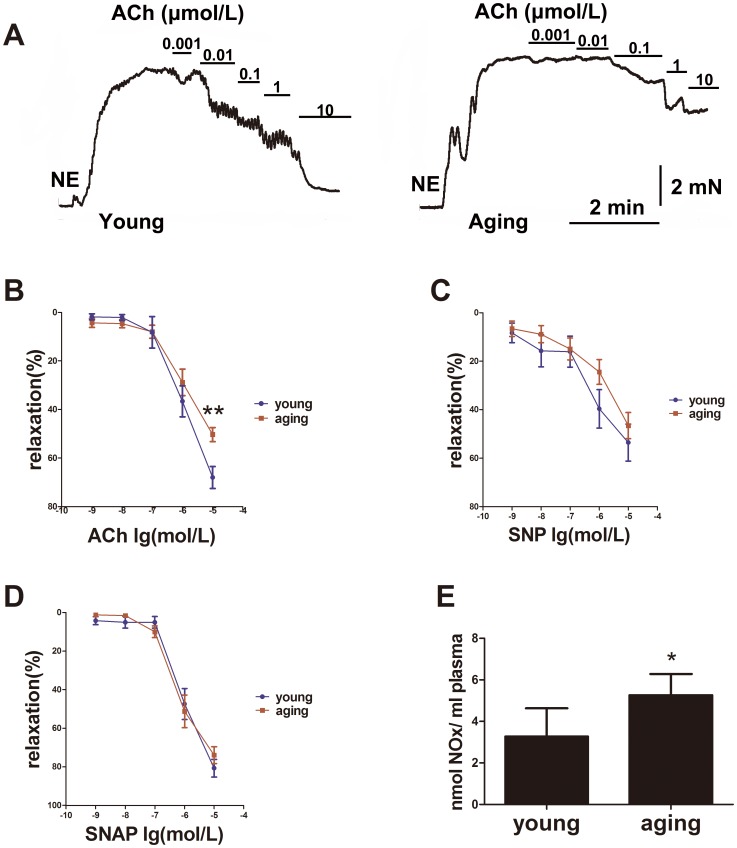
Endothelium-dependent vasorelaxation function in aging resistance arteries was impaired compared with those in young rats, while the NOx level in the serum of the aging rats was increased. (A) ACh produces dose-dependent relaxation of resistance arteries in young and aging rats. (B) ACh-induced endothelium-dependent vasorelaxation in aging resistance arteries was lower than young rats, n>6. (C, D) SNP and SNAP-induced endothelium-independent vasorelaxation remained constant, n>4. (E) NOx content in the serum of aging rats was increased compared with the young rats, n>5. **P<0.01 *vs.* young, *P<0.05 *vs.* young. Data represents the mean ± SEM in (B, C, and D), and data represents the mean ± SD in (E).

ACh (an endothelium-dependent vasodilator) relaxed the NE-induced vasoconstriction in young resistance arteries sufficiently, but the vasorelaxation responses to ACh in aging resistance arteries were impaired (Fig. 1A). Endothelium-dependent dilation in aging resistance arteries was lower than in those from young rats (*P*<0.01) (Fig. 1B). Relaxation values (% of maximal endothelium-dependent dilation induced by ACh) decreased from 68.0%±4.5% in the young animals to 50.4%±2.9% in the aging animals. However, the endothelium-independent dilation induced by SNP/SNAP in aging resistance arteries remained constant (Fig. 1C, D). It suggests an endothelium dysfunction in aging resistance arteries.

In order to detect the production of NO in aging arteries, the level of NOx in serum from rats was determined. NOx level is highly correlated with NO level in serum[20], and have been considered as a marker of vascular function. NOx level in serum from aging rats was increased compare with serum from young ones (nmol/ml; young *vs.* aging: 3.3±1.4 *vs.* 5.3±1.0, *P*<0.05) (Fig.1E), which suggested the increased NO production in aging rats' serum.

### ONOO^-^ level increased in aging resistance arteries

**Figure 2:**
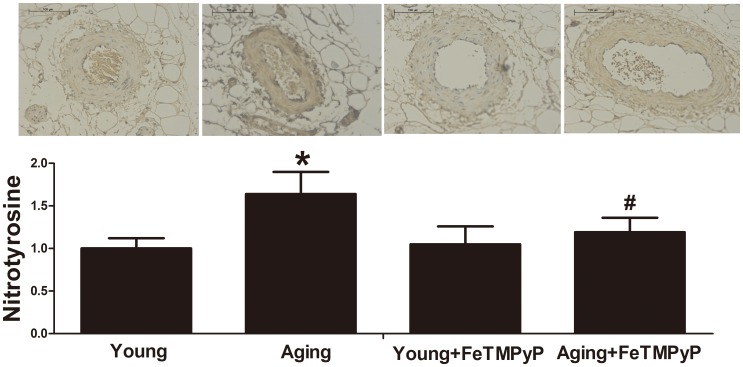
NT expression in aging resistance arteries was elevated; and FeTMPyP treatment normalized the NT expression in aging arteries, n = 3–6. *P<0.05 *vs.* young, # *P*<0.05 *vs.* aging, scale bar = 50 µm. Data represents the mean ± SD.

ONOO^-^ nitrated tyrosine on proteins to form NT. And NT is considered generally as the biomarker of the ONOO^-^ in tissues and cells [21]. To detect the NO destruction and the formation of ONOO^-^, the level of NT was determined in young and aging resistance arteries. In the present study, amounts of NT were measured using immunohistochemistry. The level of NT increased in aging rat's resistance arteries (Fig.2, *P*<0.05). Both the endothelium and vascular smooth muscle were stained immunocytochemically. It suggests an elevated ONOO^-^ level in aging resistance arteries.

### ONOO^-^ is a significant factor in aging resistance arterial vasorelaxation dysfunction

**Figure 3:**
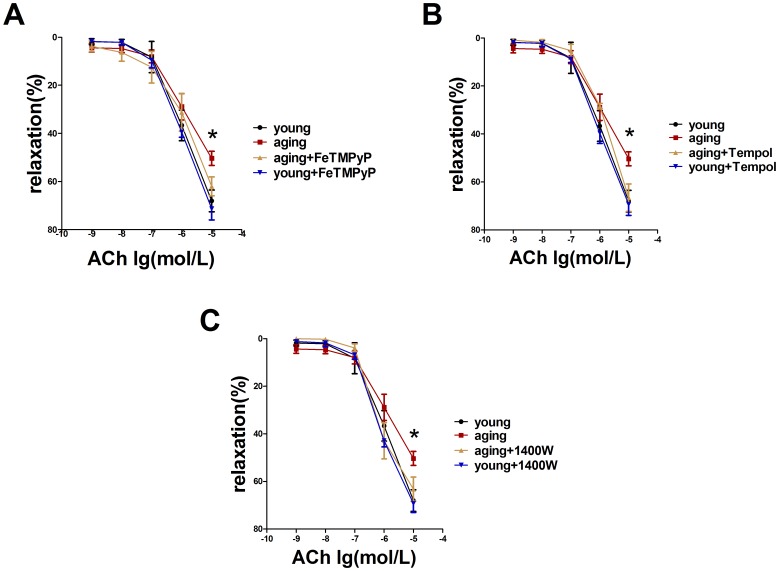
Anti-peroxynitrite treatment ameliorated endothelium-dependent vasorelaxation function in aging resistance arteries. (A) ACh-induced endothelium-dependent vasorelaxation was normalized after FeTMPyP treatment, n>6. *P<0.05 *vs.* aging+FeTMPyP. (B) ACh-induced endothelium-dependent vasorelaxation was normalized after Tempol treatment, n>4. *P<0.05 *vs.* aging+Tempol (C) ACh-induced endothelium-dependent vasorelaxation was normalized after 1400W treatment, n>4. *P<0.05 *vs.* aging+1400W. Data represents the mean ± SEM.

In order to certify the role of ONOO^-^ in aging-related resistance arterial vasorelaxation dysfunction, ONOO^-^ scavenger (FeTMPyP, 10^−5^ mol/L) was used to inhibit ONOO^-^ in aging resistance arteries. Vasorelaxation responses to ACh were normalized after ONOO^-^ scavenger preincubation in aging resistance arteries (Fig. 3A; *P*<0.05). FeTMPyP treatment increased relaxation values from 50.4%±2.9% to 61.5%±3.5% in the aging animals. ONOO^-^ is the product of the interaction between**^.^**O_2_^-^ and NO. The present study inhibited the**^.^**O_2_^-^ and iNOS by Tempol (10^-4^ mol/L) and 1400W (10^−6^ mol/L), respectively. Inhibition of either**^.^**O_2_^-^ or iNOS ameliorated the vasorelaxation function in aging resistance arteries (aging+1400W/aging+Tempol *vs.* aging: 63.3%±5.1%/66.6%±5.9% *vs.* 50.4%±2.9%; Fig.3 B, C, *P*<0.05). The inhibitor (FeTMPyP, 1400W or Tempol) treatment showed no significant effect on vasorelaxation of young resistance arteries. It suggests that ONOO^-^ is a detrimental factor in aging-related resistance arterial vasorelaxation dysfunction.

### Treatment with ONOO^-^ scavenger (FeTMPyP) ameliorated NO-sGC-cGKs signaling pathway

**Figure 4:**
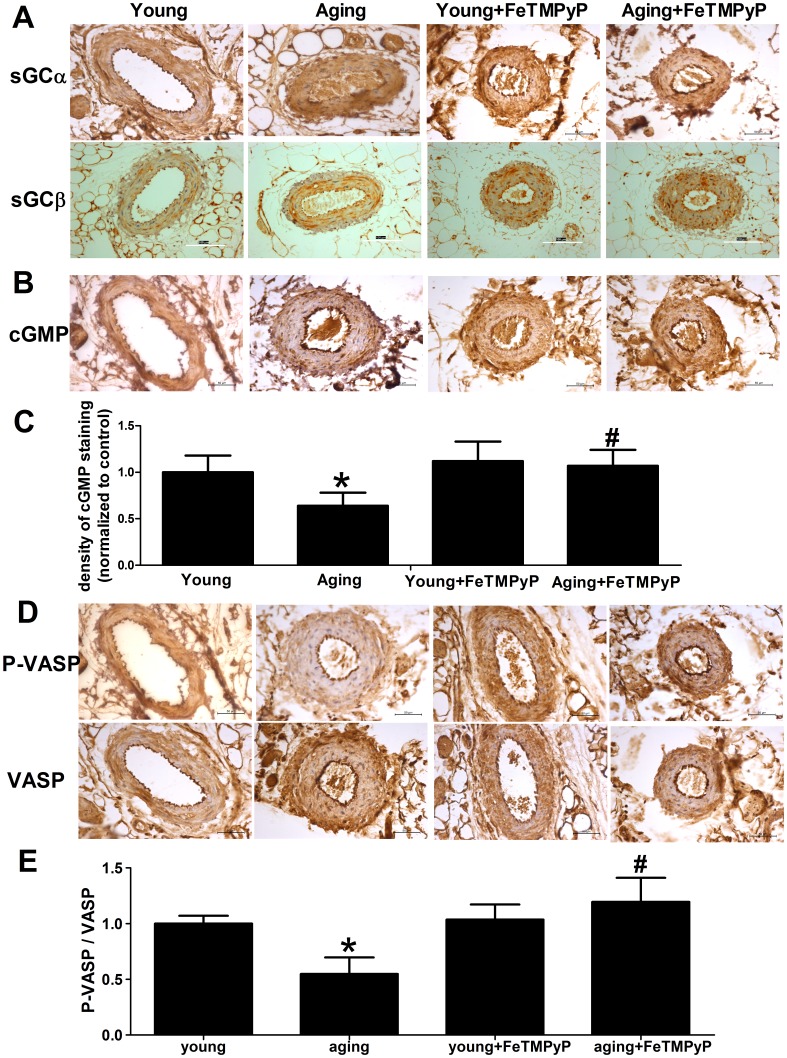
sGC expression remained constant, but cGMP and P-VASP/VASP ratio was decreased in aging resistance arteries; and FeTMPyP treatment reversed aging-related downregulation of the P-VASP/VASP ratio. (A, B, D) sGC α1, sGC β1, cGMP, VASP and P-VASP immunostaining in resistance arteries. Expression of sGC α1 and sGC β1 in young and aging resistance arteries was not significant different. (C, E) Densitometry analysis of the cGMP level and P-VASP/VASP ratio in resistance arteries. Senescence decreased the cGMP and P-VASP/VASP ratio, and which was reversed by the FeTMPyP treatment in vivo. n = 3-6. *P<0.05 *vs.* young, # *P*<0.05 *vs.* aging, scale bar = 50 µm. Data represents the mean ± SD.

To further identify the mechanism of ONOO^-^-induced vasorelaxation dysfunction in aging resistance arteries, the NO-sGC-cGKs pathway was determined. sGC is the cytoplasmic receptor of NO, which plays an important role in NO-sGC-cGKs signaling. Expression of sGC was detected by immunohistochemistry. Expression of sGC α1 and sGC β1 subunit was unaltered in aging resistance arteries compared with young resistance arteries (Fig. 4A). And FeTMPyP treatment showed no effect on sGC subunits expression in resistance arteries. When the NO-sGC-cGKs signaling is activated, cGMP level increases and cGKs phosphorylate VASP at serine 239 to form P-VASP. cGMP level decreased in aging resistance arteries, which was reversed by FeTMPyP treatment (Fig.4B, C; *P*<0.05). The P-VASP/VASP ratio is a biochemical marker for NO-sGC-cGKs signaling in vascular tissues. The P-VASP/VASP ratio was significantly diminished in aging resistance arteries (Fig.4D, E; *P*<0.05). ONOO^-^ scavenger treatment elevated P-VASP/VASP ratio in aging resistance arteries (Fig. 4B, C; *P*<0.05) but not the young ones. And ONOO^-^ scavenger treatment suppressed the expression of NT in aging resistance arteries (Fig.2). The present results suggest that ONOO^-^ is involved with the NO-sGC-cGKs signaling pathway dysfunction in aging resistance arteries.

## Discussion

The present study confirms that vasorelaxation function decreased in aging resistance arteries. And the elevated ONOO^-^ level is responsible for the vasorelaxation dysfunction in aging resistance arteries. Moreover, the present study suggests that ONOO^-^ markedly impaired the NO-sGC-cGKs signaling, which may be a significant mechanism for aging-related resistance arterial vasorelaxation dysfunction.

The homeostasis of resistance arteries plays an important role in the regulation of blood pressure [22]. Evidence exists that endothelium-dependent vasorelaxation is impaired in the omental arteries from aging human without hypertension [23]. In the present study, the blood pressure was unaltered in aging rats compared with young ones (table 1). And the dilation responses to ACh, an endothelium-dependent vasodilator, were decreased in resistance arteries isolated from aging animals. It suggests that even the aging animals without hypertension have the endothelium-dependent vasorelaxation dysfunction. The extensive existence of endothelium dysfunction in aging animals may explain the susceptibility of vascular diseases during aging. And the present study had measured dilation responses to two kinds of NO donors, SNP and SNAP, in resistance arteries. SNP is an enzymatic-dependent NO donor. The process of SNP mediated NO release requires the thiols in tissues [24]. SNAP is an enzymatic-independent NO donor, and the progress of SNAP mediated NO release is spontaneous in the water [25]. Nevertheless, dilation responses to SNP and SNAP were unaltered in aging resistance arteries compared with young resistance arteries. It suggests that the vascular smooth muscle in aging resistance arteries has a normal function.

**Table 1:** The weight, heart rates, systolic pressures and diastolic pressures of animals.

	Young	Aging	Aging+FeTMPyP
**Wight(g)**	251.7±16.0	693.0±68.4**	661.3±64.6**
**Heart Rate(time/min)**	359.7±11.4	363.1±13.2	366.4±9.0
**Systolic Pressure(mmgH)**	105.4±5.7	104.3±6.1	100.9±10. 7
**Diastolic Pressure (mmgH)**	80.4±5.0	80.8±4.1	79.0±8.7

**P<0.01 *vs.* young.

NO is one of the endothelium-derived relaxing factors (EDRF). The decreased NO bioavailability is involved in many aging-related vascular dysfunction. In the present study, the level of NOx was elevated in the serum from aging rats. NOx is generally considered as the level of vascular NO and its in vivo metabolic products. Increased level of NOx suggests the elevated NO production in aging vasculature. Most of the NO in the arteries is produced from endothelial nitric oxide synthase (eNOS) and iNOS. eNOS is the major player in NO production in the young arteries, and eNOS-derived NO has been considered as the fundamental mediator of vascular homeostasis. However, the expression of eNOS seems to be controversial in the aging arteries. Both the increased [26] and declined expression [27] of eNOS has been reported in the previous studies. However, the activity of eNOS is impaired in aging arteries [28], which suggests that the eNOS does not appear to be responsible for the increased NO production in aging serum. On the other hand, iNOS expression is increased in aging arteries [29]. And the NO production ability of iNOS is much higher than eNOS. Therefore, we presume that increased iNOS expression is the main effecting factors in the elevated NO production in aging arteries.

Increased superoxide generation is involved in the NO destruction. Considerable evidence exists that the increased oxidative stress in aging arteries is associated with elevated superoxide production [30]. It is the consequence of increased NADPH oxidase expression and the anti-oxidative system dysfunction. Increased**^.^**O_2_^-^ reacts with NO, and this reaction produces ONOO^-^ [31]. This reaction inactivates NO and the production ONOO^-^ has been indicated to have a destructive effect on vascular function. Our preliminary study revealed that the level of NT (biomarker of ONOO^-^) was increased in aging aorta, which was suppressed by inhibition of iNOS [32]. It reveals that iNOS derived NO may be inactivated in aging arteries. In the present study, the expression of NT was increased in aging resistance arteries. It suggests the NO destruction and the ONOO^-^ production in the aging resistance arteries.

Studies have shown that increased ONOO^-^ is associated with vasorelaxation dysfunction in aging arteries, such as the aorta [33] and the coronary artery [34]. And in our preliminary study, the vasorelaxation function of aging aorta was ameliorated after suppressing the level of ONOO^-^ by inhibition the iNOS expression [32]. In the present study, ONOO^-^ and its source (**^.^**O_2_^-^ and iNOS) were inhibited respectively to demonstrate the role of ONOO^-^ in the vasorelaxation dysfunction in aging resistance arteries. FeTMPyP is the specific scavenger of ONOO^-^. FeTMPyP treatment ameliorated ACh-induced dilation in aging resistance arteries. In addition, either Tempol (**^.^**O_2_^-^ scavenger) or 1400W (iNOS inhibitor) preincubation normalized vasorelaxation responses to ACh in aging resistance arteries. It suggests that ONOO^-^ is involved in the vasorelaxation dysfunction in aging resistance arteries.

The NO-sGC-cGKs pathway, by which NO reduces vascular tone, is crucial to the regulation of vasorelaxation in resistance arteries. sGC is the cytoplasmic receptor of NO, which plays an important role in NO-derived vasorelaxation. NO binds to the heme cofactor of sGC and enhances the production of cGMP, which induces the activation of cGMP-dependent protein kinases (cGKs) and vasorelaxation [35]. Heterodimeric sGC consists of α and β subunits, and expresses in most tissues. Vasodilatation is mediated primarily by the α1β1 isoform in arteries [36]. Deletion of either α1 [37] or β1 [38] subunit in mice exhibited elevated blood pressure and dysfunction in smooth muscle contractility. It is reported that the decreased expression of sGC subunits was one of the mechanisms for vasodilatation dysfunction in aging aorta [39]. The reduced expression of sGC in the aorta is mediated by downregulation of the human-antigen R (HuR) [40]. And HuR is mRNA-binding protein, which stabilizes the sGC subunits mRNA at the posttranscriptional level. However, in the present study, expression of both the sGC α1 and sGC β1 was not significantly different between aging and young resistance arteries. It suggests that the mechanism of aging related vascular impairment in various types of arteries is different. cGMP is the production of sGC. In the present study, cGMP level was downregulated in aging resistance arteries, and FeTMPyP elevated cGMP level. We consider two important possibilities regarding the relationship between the unchanged sGC expression and decreased cGMP in aging resistance arteries. The first possibility is that sGC itself may be inactivited by ONOO^-^ in aging resistance arteries. Evidence exists that ONOO^-^ alters the activity of proteins by nitrated the tyrosine residues[41]. And the results of the present study showed that the total NT expression in the aging resistance arteries is increased. The second possibility deserves consideration is that the production of ONOO^-^ consumes NO and reduces the activity of sGC. In the present study, Nox level is increased in serum from aging rats. NOx is a marker of NO production but not NO bioavailability in aging arteries. The elevated NO production may be consumed by reactive oxygen species in aging resistance arteries and leads to sGC inactivity.

The NO-sGC-cGKs pathway plays a critical role in vascular tone maintaining. The present study suggests that impaired NO-sGC-cGKs pathway may be responsible for the vasorelaxation dysfunction in aging arteries. When NO-sGC-cGKs pathway is activated, cGKs phosphorylate VASP at serine 239 [42]. The P-VASP/VASP ratio is considered a biochemical marker to monitor the NO-sGC-cGKs signaling pathway [43]. In the present study, P-VASP/VASP ratio was declined in aging resistance arteries, which indicated the NO-sGC-cGKs pathway inhibition. FeTMPyP treatment in vivo down regulated the expression of NT and reversed the P-VASP/VASP ratio in aging resistance arteries. It suggests that the increased ONOO^-^ may be an important cause of vasorelaxation dysfunction in aging resistance arteries. In addition, it is reported that ONOO^-^ may be involved in numerous physiological processes in vasorelaxation. For example, ONOO^-^ up regulated the activity of prostaglandin endoperoxide H_2_ synthase (PGHS) and down regulated the activity of prostacyclin-synthase (PGI2-synth), suggesting that ONOO^-^ is involved with the disorder of the synthesis of prostaglandins in aging arteries [44]; and ONOO^-^ impaired the endothelium-derived hyperpolarizing factor (EDHF) induced vasorelaxation by injury of the potassium channels, such as large-conductance Ca^2+^-activated K^+^ channels (BK_Ca_) [45], small-conductance K_Ca_ (SK) and intermediate-conductance K_Ca_ (IK) [46]. In the case of an elevated level of ONOO^-^ in aging resistance arteries, vasorelaxation dysfunction induced by ONOO^-^ may be underlying mechanisms of vascular tone maintaining dysfunction in aging resistance arteries.

In the present study, the NT level was decreased incompletely after FeTMPyP treatment, but p-VASP/VASP value was fully ameliorated. The possibility that warrants consideration is that NT is the production of ONOO^-^ nitrated proteins but not ONOO^-^ itself. FeTMPyP treatment may scavenge ONOO^-^ sufficiently and ameliorated the p-VASP/VASP value in the aging resistance arteries, but the nitration of tyrosine on proteins has not been removed completely after FeTMPyP treatment.

In conclusion, the present study demonstrates that ONOO^-^ is responsible for relaxation dysfunction in aging resistance arteries, which is related to the impairment of the NO-sGC-cGKs pathway, suggesting that the therapeutic interventions which inhibit ONOO^-^ or its sources may improve age-related resistance arterial vasodilatation dysfunction.

## References

[1] Lesniewski LA, Durrant JR, Connell ML, Henson GD, Black AD, et al (2011) Aerobic exercise reverses arterial inflammation with aging in mice. Am J Physiol Heart Circ Physiol 301: H1025–H1032. 21622824 10.1152/ajpheart.01276.2010 PMC3191071

[2] Moreau P, D'Uscio LV, Luscher TF (1998) Structure and reactivity of small arteries in aging. Cardiovasc Res 37: 247–253. 9539880 10.1016/s0008-6363(97)00225-3

[3] Toda N, Toda H (2011) Coronary hemodynamic regulation by nitric oxide in experimental animals: recent advances. Eur J Pharmacol 667: 41–49. 21741964 10.1016/j.ejphar.2011.06.028

[4] Angulo J, Vallejo S, El AM, Garcia-Septiem J, Sanchez-Ferrer CF, et al (2012) Age-related differences in the effects of alpha and gamma peroxisome proliferator-activated receptor subtype agonists on endothelial vasodilation in human microvessels. Exp Gerontol 47: 734–740. 22776133 10.1016/j.exger.2012.06.014

[5] Marasciulo FL, Montagnani M, Potenza MA (2006) Endothelin-1: the yin and yang on vascular function. Curr Med Chem 13: 1655–1665. 16787211 10.2174/092986706777441968

[6] Idris-Khodja N, Schini-Kerth V (2012) Thymoquinone improves aging-related endothelial dysfunction in the rat mesenteric artery. Naunyn Schmiedebergs Arch Pharmacol 385: 749–758. 22526469 10.1007/s00210-012-0749-8

[7] Hong CW, Kim YM, Pyo H, Lee JH, Kim S, et al (2013) Involvement of inducible nitric oxide synthase in radiation-induced vascular endothelial damage. J Radiat Res. 10.1093/jrr/rrt066 PMC3823786 23704776

[8] Cernadas MR, Sanchez DML, Garcia-Duran M, Gonzalez-Fernandez F, Millas I, et al (1998) Expression of constitutive and inducible nitric oxide synthases in the vascular wall of young and aging rats. Circ Res 83: 279–286. 9710120 10.1161/01.res.83.3.279

[9] Zhou X, Bohlen HG, Unthank JL, Miller SJ (2009) Abnormal nitric oxide production in aged rat mesenteric arteries is mediated by NAD(P)H oxidase-derived peroxide. Am J Physiol Heart Circ Physiol 297: H2227–H2233. 19783779 10.1152/ajpheart.00325.2009 PMC2793129

[10] Tao L, Liu HR, Gao E, Teng ZP, Lopez BL, et al (2003) Antioxidative, antinitrative, and vasculoprotective effects of a peroxisome proliferator-activated receptor-gamma agonist in hypercholesterolemia. Circulation 108: 2805–2811. 14610009 10.1161/01.CIR.0000097003.49585.5E

[11] Cabassi A, Binno SM, Tedeschi S, Ruzicka V, Dancelli S, et al (2014) Low Serum Ferroxidase I Activity is Associated with Mortality in Heart Failure and Related to Both Peroxynitrite-Induced Cysteine Oxidation and Tyrosine Nitration of Ceruloplasmin. Circ Res. 10.1161/CIRCRESAHA.114.302849 24687133

[12] Daiber A, Daub S, Bachschmid M, Schildknecht S, Oelze M, et al (2013) Protein tyrosine nitration and thiol oxidation by peroxynitrite-strategies to prevent these oxidative modifications. Int J Mol Sci 14: 7542–7570. 23567270 10.3390/ijms14047542 PMC3645702

[13] James MA, Tullett J, Hemsley AG, Shore AC (2006) Effects of aging and hypertension on the microcirculation. Hypertension 47: 968–974. 16505195 10.1161/10.1161/01.HYP.0000209939.05482.61

[14] Prisby RD, Ramsey MW, Behnke BJ, Dominguez JN, Donato AJ, et al (2007) Aging reduces skeletal blood flow, endothelium-dependent vasodilation, and NO bioavailability in rats. J Bone Miner Res 22: 1280–1288. 17451371 10.1359/jbmr.070415

[15] Peter BF, Lidington D, Harada A, Bolz HJ, Vogel L, et al (2008) Role of sphingosine-1-phosphate phosphohydrolase 1 in the regulation of resistance artery tone. Circ Res 103: 315–324. 18583713 10.1161/CIRCRESAHA.108.173575 PMC2746908

[16] Palomares SM, Gardner-Morse I, Sweet JG, Cipolla MJ (2012) Peroxynitrite decomposition with FeTMPyP improves plasma-induced vascular dysfunction and infarction during mild but not severe hyperglycemic stroke. J Cereb Blood Flow Metab 32: 1035–1045. 22373645 10.1038/jcbfm.2012.14 PMC3367219

[17] Lesniewski LA, Zigler ML, Durrant JR, Nowlan MJ, Folian BJ, et al (2013) Aging compounds western diet-associated large artery endothelial dysfunction in mice: prevention by voluntary aerobic exercise. Exp Gerontol 48: 1218–1225. 23954368 10.1016/j.exger.2013.08.001 PMC3840721

[18] Staehr M, Madsen K, Vanhoutte PM, Hansen PB, Jensen BL (2011) Disruption of COX-2 and eNOS does not confer protection from cardiovascular failure in lipopolysaccharide-treated conscious mice and isolated vascular rings. Am J Physiol Regul Integr Comp Physiol 301: R412–R420. 21543636 10.1152/ajpregu.00823.2010

[19] Yan Z, Liang F, Guo L, Wang J, Wang XL, et al (2010) Myeloperoxidase increased cardiomyocyte protein nitration in mice subjected to nonlethal mechanical trauma. Biochem Biophys Res Commun 393: 531–535. 20153732 10.1016/j.bbrc.2010.02.049

[20] Sastry KV, Moudgal RP, Mohan J, Tyagi JS, Rao GS (2002) Spectrophotometric determination of serum nitrite and nitrate by copper-cadmium alloy. Anal Biochem 306: 79–82. 12069417 10.1006/abio.2002.5676

[21] Seimetz M, Parajuli N, Pichl A, Veit F, Kwapiszewska G, et al (2011) Inducible NOS inhibition reverses tobacco-smoke-induced emphysema and pulmonary hypertension in mice. Cell 147: 293–305. 22000010 10.1016/j.cell.2011.08.035

[22] Zhang H, Fisher SA (2007) Conditioning effect of blood flow on resistance artery smooth muscle myosin phosphatase. Circ Res 100: 730–737. 17293476 10.1161/01.RES.0000260189.38975.35

[23] Leocadio Rodrı Guez-Man As ME, Susana Vallejo PLPR, Roberto Petidier MMJN, Marta Castro CGM, Nchez-Ferrer CPAC, et al. (2009) Endothelial dysfunction in aged humans is related with oxidative stress and vascular inflammation. aging cell. 10.1111/j.1474-9726.2009.00466.x 19245678

[24] Wang PG, Xian M, Tang X, Wu X, Wen Z, et al (2002) Nitric oxide donors: chemical activities and biological applications. Chem Rev 102: 1091–1134. 11942788 10.1021/cr000040l

[25] Stibingerova A, Velvarska H, Kynclova K, Marounkova B, Spundova M, et al (2009) Lipoamide dehydrogenase and diaphorase catalyzed conversion of some NO donors to NO and reduction of NO scavenger 2-phenyl-4,4,5,5-tetramethylimidazoline-1-oxyl-3-oxide (PTIO). Gen Physiol Biophys 28: 384–390. 20097961 10.4149/gpb_2009_04_384

[26] Goettsch W, Lattmann T, Amann K, Szibor M, Morawietz H, et al (2001) Increased expression of endothelin-1 and inducible nitric oxide synthase isoform II in aging arteries in vivo: implications for atherosclerosis. Biochem Biophys Res Commun 280: 908–913. 11162610 10.1006/bbrc.2000.4180

[27] Yoon HJ, Cho SW, Ahn BW, Yang SY (2010) Alterations in the activity and expression of endothelial NO synthase in aged human endothelial cells. Mech Ageing Dev 131: 119–123. 20064546 10.1016/j.mad.2009.12.010

[28] Yoon HJ, Cho SW, Ahn BW, Yang SY (2010) Alterations in the activity and expression of endothelial NO synthase in aged human endothelial cells. Mech Ageing Dev 131: 119–123. 20064546 10.1016/j.mad.2009.12.010

[29] Cau SB, Carneiro FS, Tostes RC (2012) Differential modulation of nitric oxide synthases in aging: therapeutic opportunities. Front Physiol 3: 218. 22737132 10.3389/fphys.2012.00218 PMC3382417

[30] Fleenor BS, Seals DR, Zigler ML, Sindler AL (2012) Superoxide-lowering therapy with TEMPOL reverses arterial dysfunction with aging in mice. Aging Cell 11: 269–276. 22168264 10.1111/j.1474-9726.2011.00783.x PMC3409251

[31] Stavniichuk R, Shevalye H, Lupachyk S, Obrosov A, Groves JT, et al (2014) Peroxynitrite and protein nitration in the pathogenesis of diabetic peripheral neuropathy. Diabetes Metab Res Rev. 10.1002/dmrr.2549 PMC4177961 24687457

[32] Tian J, Yan Z, Wu Y, Zhang SL, Wang K, et al (2010) Inhibition of iNOS protects endothelial-dependent vasodilation in aged rats. Acta Pharmacol Sin 31: 1324–1328. 20835265 10.1038/aps.2010.111 PMC4012905

[33] Radovits T, Seres L, Gero D, Lin LN, Beller CJ, et al (2007) The peroxynitrite decomposition catalyst FP15 improves ageing-associated cardiac and vascular dysfunction. Mech Ageing Dev 128: 173–181. 17116320 10.1016/j.mad.2006.09.005

[34] Csiszar A, Ungvari Z, Edwards JG, Kaminski P, Wolin MS, et al (2002) Aging-induced phenotypic changes and oxidative stress impair coronary arteriolar function. Circ Res 90: 1159–1166. 12065318 10.1161/01.res.0000020401.61826.ea

[35] Nardi GM, Scheschowitsch K, Ammar D, de Oliveira SK, Arruda TB, et al (2014) Neuronal Nitric Oxide Synthase and Its Interaction With Soluble Guanylate Cyclase Is a Key Factor for the Vascular Dysfunction of Experimental Sepsis. Crit Care Med. 10.1097/CCM.0000000000000301 24717470

[36] Derbyshire ER, Marletta MA (2012) Structure and regulation of soluble guanylate cyclase. Annu Rev Biochem 81: 533–559. 22404633 10.1146/annurev-biochem-050410-100030

[37] Mergia E, Friebe A, Dangel O, Russwurm M, Koesling D (2006) Spare guanylyl cyclase NO receptors ensure high NO sensitivity in the vascular system. J Clin Invest 116: 1731–1737. 16614755 10.1172/JCI27657 PMC1435723

[38] Friebe A, Mergia E, Dangel O, Lange A, Koesling D (2007) Fatal gastrointestinal obstruction and hypertension in mice lacking nitric oxide-sensitive guanylyl cyclase. Proc Natl Acad Sci U S A 104: 7699–7704. 17452643 10.1073/pnas.0609778104 PMC1863512

[39] Kloss S, Bouloumie A, Mulsch A (2000) Aging and chronic hypertension decrease expression of rat aortic soluble guanylyl cyclase. Hypertension 35: 43–47. 10642273

[40] Kloss S, Rodenbach D, Bordel R, Mulsch A (2005) Human-antigen R (HuR) expression in hypertension: downregulation of the mRNA stabilizing protein HuR in genetic hypertension. Hypertension 45: 1200–1206. 15883232 10.1161/01.HYP.0000165674.58470.8f

[41] Martinez-Ruiz A, Cadenas S, Lamas S (2011) Nitric oxide signaling: classical, less classical, and nonclassical mechanisms. Free Radic Biol Med 51: 17–29. 21549190 10.1016/j.freeradbiomed.2011.04.010

[42] Bucci M, Papapetropoulos A, Vellecco V, Zhou Z, Zaid A, et al (2012) cGMP-dependent protein kinase contributes to hydrogen sulfide-stimulated vasorelaxation. PLoS One 7: e53319. 23285278 10.1371/journal.pone.0053319 PMC3532056

[43] Oelze M, Mollnau H, Hoffmann N, Warnholtz A, Bodenschatz M, et al (2000) Vasodilator-stimulated phosphoprotein serine 239 phosphorylation as a sensitive monitor of defective nitric oxide/cGMP signaling and endothelial dysfunction. Circ Res 87: 999–1005. 11090544 10.1161/01.res.87.11.999

[44] Schildknecht S, Ullrich V (2009) Peroxynitrite as regulator of vascular prostanoid synthesis. Arch Biochem Biophys 484: 183–189. 18983814 10.1016/j.abb.2008.10.023

[45] Liu Y, Terata K, Chai Q, Li H, Kleinman LH, et al (2002) Peroxynitrite inhibits Ca2+-activated K+ channel activity in smooth muscle of human coronary arterioles. Circ Res 91: 1070–1076. 12456494 10.1161/01.res.0000046003.14031.98

[46] Cheng Z, Jiang X, Kruger WD, Pratico D, Gupta S, et al (2011) Hyperhomocysteinemia impairs endothelium-derived hyperpolarizing factor-mediated vasorelaxation in transgenic cystathionine beta synthase-deficient mice. Blood 118: 1998–2006. 21653942 10.1182/blood-2011-01-333310 PMC3158725

